# Does erythropoietin slow progression of chronic kidney disease?

**DOI:** 10.12861/jrip.2013.25

**Published:** 2013-06-01

**Authors:** Hamid Nasri, Ali Ghorbani

**Affiliations:** ^1^Department of Nephrology, Division of Nephropathology, Isfahan University of Medical Sciences, Isfahan, Iran; ^2^Department of Nephrology, Golestan Hospital, Ahvaz Jundishapur University of Medical Sciences, Ahvaz, Iran

**Keywords:** Erythropoietin, Erythropoiesis, Kidney protection, Chronic kidney disease

**Implication for health policy/practice/research/medical education:**

The initiation of erythropoietin therapy for the purpose of renoprotection may need to be sooner than that for erythropoiesis, because erythropoietin may attenuate renal fibrosis through macrophage adjustment and endothelial cell protection by other uncertain mechanisms. Although, agents re-establishing the initial function of renal erythropoietin-producing cells could defer kidney fibrosis, however more studies should be carried out to determine the cellular target of erythropoietin in kidney and developinga new erythropoietin derivative for renal care.

Erythropoietin improves anemia in almost all patients with chronickidney disease (CKD) (1,2). Anemia seems to be an indicator of tissue hypoxia which deteriorates kidney damage in CKD (1-3). Erythropoietin provokes red blood cell maturation in bone marrow and accentuates erythropoiesis (3,4). It is a glycoprotein and a member of class I cytokines (3,4). Peritubular interstitial fibroblasts in the renal cortex and outer medulla release most part of erythropoietin (3-5). The amount of oxygen supply to the tissues appears to control erythropoietin synthesis in a feedback pathway. Hypoxia induces a factor that regulates the renal erythropoietin gene transcription, which in turn, controls the productionof erythropoietin (3-5). Renal fibrosis is the final common event in all CKD types with different etiologies (5,6). Persistent inflammation and transition of pericytes to myofibroblast causes kidney fibrosis and lesser amount of erythropoietin production (4-7). To date, administration of erythropoietin has had a significant impact on anemia improvement and reduced hypoxic tissue damage (6-9). New upcoming data suggest renoprotective potentials of erythropoietin. Recently, renoprotective effect of erythropoietin has become revealed which is not related to its erythropoetic properties (10-12). Different studies have shown renal protective effect of erythropoietin in acute kidney injury.

In a trial, we tested the hypothesis that erythropoietin protects renal tubular cells, enrolling 40 male rats. We showed that erythropoietin prevented the kidneys from acute kidney injury. Also, we found that administration of erythropoietin along with gentamicin reduces renal damage comparing control group. As well; erythropoietin was also effective, when it was administered after the occurrence of gentamicin-induced tubular damage. This revealed that erythropoietin was still effective after installation of renal damage (13). Therefore, erythropoietin seems to be a promising renal protective agent against nephrotoxic tubular damage caused by gentamicin or other aminoglycosides (1-6,14). Recent studies have unveiled the cellular mechanism of renal erythropoietin synthesis and the following events leading to renal fibrosis (2-7,14-16). Interestingly, fibroblasts from damaged tubular epithelial cells have no significant contribution in renal fibrosis, but renal erythropoietin-producing cells, originating from neural crests, differentiate into myofibroblasts in long time exposure to inflammation. It seems that they are involved in renal fibrosis (6,8,17,18). Indeed, nearly all myofibroblasts that express α-smooth muscle act in originates from the renal erythropoietin-producingcells; they are normally peritubular interstitial fibroblastic cells expressing neural cell marker genes but they do not express α-smooth muscle actin. Macrophages and myofibroblasts are dominant cells causing kidney fibrosis. Macrophages can be differentiated to phenotype M1 (classically activated) or M2 (wound healing) regarding to the distinctive cytokine production (1-8,14-17). While, erythropoietin can disconnect macrophages by abolishing the activity of NF-κB*, in vivo* macrophage regulation could be one of the mechanisms that explain the antifibrotic properties of erythropoietin in CKD (13-18). These important findings define the missing link in CKD between renal fibrosis and anemia (14-18). Some recent studies have indicated the improvement of renal function in CKD following administration of erythropoietin (12-18). Different notable evidences imply some benefits of erythropoietin other than the improvement of anemia such as the pleiotropic effects on the cardiovascular system and on the kidney (11-17). Clinical evidences suggest the erythropoietin renoprotective potential in patients with CKD, however more clinical trials are needed to clarify the time of initiation of erythropoietin treatment and the optimum dose of erythropoietin for reduction of disease progression in patients with CKD. The initiation of erythropoietin therapy for the purpose of renoprotection may need to be sooner than that for erythropoiesis, because erythropoietin may attenuate renal fibrosis through macrophage adjustment and endothelial cell protection by other uncertain mechanisms (1-9). Although, agents re-establishing the initial function of renal erythropoietin-producing cells could defer kidney fibrosis, more studies should be carried out to determine the cellular target of erythropoietin in kidney and developing a new erythropoietin derivative for renal care (15-18).

## Authors’ contributions

HN and AG wrote the manuscript equally.

## Conflict of interests

The author declared no competing interests.

## Ethical considerations

Ethical issues (including plagiarism, informed consent, misconduct, double publication and redundancy) have been completely observed by the authors.

## Funding/Support

None declared.

## References

[1] Gianella P, Martin PY, Stucker F (2013). Management of renal anemia in 2013. Rev Med Suisse.

[2] (2006). Association of serum leptin with anemia in maintenance hemodialysis patients. Saudi J Kidney Dis Transpl.

[3] Bahlmann FH, Kielstein JT, Haller H, Fliser D (2007). Erythropoietin andprogression of CKD. Kidney Int Suppl.

[4] Tanaka T, Nangaku M (2012). Recent advances and clinical application of erythropoietin and erythropoiesis-stimulating agents. Xp Cell Res.

[5] Babitt JL, Lin HY (2012). Mechanisms of anemia in CKD. J Am Soc Nephrol.

[6] Chang FC, Chou YH, Chen YT, Lin SL (2012). Novel insights into pericyte-myofibroblast transition and therapeutic targets in renal fibrosis. J Formos Med Assoc.

[7] Rafieian-Kopaei M, Nasri H. Comment on: Is the renoprotective effect of erythropoietin in chronic kidney disease a myth? J Formos Med Assoc 2013. 10.1016/j.jfma.2013.05.006 23850010

[8] Lin SL, Duffield JS (2012). Macrophages in kidney injury and repair. Acta Nephrol.

[9] Gouva C, Nikolopoulos P, Ioannidis JP, Siamopoulos KC (2004). Treating anemia early in renal failure patients slows the decline of renal function: a randomized controlled trial. Kidney Int.

[10] Rafieian-Kopaei M, Nasri H. Re: Erythropoietin Ameliorates Oxidative Stress and Tissue Injury following Renal Ischemia/Reperfusion in Rat Kidney and Lung. Med Princ Pract 2013. 10.1159/000350842 PMC5586822 23711458

[11] Brines M, Grasso G, Fiordaliso F, Sfacteria A, Ghezzi P, Fratelli M (2004). Erythropoietin mediates tissue protection through an erythropoietin and common beta-subunit heteroreceptor. Proc Natl Acad Sci USA.

[12] Joyeux-Faure M (2007). Cellular protection by erythropoietin: new therapeutic implications?. J Pharmacol Exp Ther.

[13] Rafieian-Kopaei M, Nasri H, Nematbakhsh M, Baradaran A, Gheissari A, Rouhi H (2012). Erythropoietin ameliorates gentamicin-induced renal toxicity: A biochemical and histopathological study. J Nephropathology.

[14] Maxwell PH, Ferguson DJ, Nicholls LG (1997). The interstitial response to renal injury: fibroblast-like cells show phenotypic changes and have reduced potential for erythropoietin gene expression. Kidney Int.

[15] Asada N, Takase M, Nakamura J (2011). Dysfunction of fibroblasts of extrarenal origin underlies renal fibrosis and renal anemia in mice. J Clin Invest.

[16] Sautina L, Sautin Y, Beem E, Zhou Z, Schuler A, Brennan J (2010). Induction of nitric oxide by erythropoietin is mediated by the β common receptor and requires interaction with VEGF receptor 2. Blood.

[17] Moore E, Bellomo R (2011). Erythropoietin (EPO) in acute kidney injury. Ann Intensive Care.

[18] Baradaran A, Nasri H (2013). Re: effect of erythropoietin on kidney allograft survival: early use after transplantation. Iran J Kidney Dis.

